# Perceptions and Bias of Small Business Leaders in Employing People with Different Types of Disabilities

**DOI:** 10.1007/s10926-024-10201-2

**Published:** 2024-05-13

**Authors:** Nanette Goodman, Samantha Deane, Fitore Hyseni, Michal Soffer, Gary Shaheen, Peter Blanck

**Affiliations:** 1 Burton Blatt Institute, Syracuse University, Syracuse, NY USA; 2 Rutgers University School of Management and Labor Relations, New Brunswick, NJ USA; 3 School of Social Work, Faculty of Social Welfare & Health Sciences, University of Haifa, Haifa, Israel

**Keywords:** Employer attitutudes, Disability employment, Small business, Disability discrimination

## Abstract

**Purpose:**

Despite existing employment-related legislation and governmental programs, people with disabilities continue to face significant barriers to competitive employment. These obstacles are partially due to biases among employers regarding the contributions of people with disabilities and perceptions about accommodation costs, which can affect their hiring decisions. Existing research on employment barriers and facilitators often treats people with disabilities homogenously and focuses mainly on large companies. This study helps to fill these gaps by exploring the motivations and challenges small employers face when hiring people with disabilities and how their attitudes and willingness to hire vary based on disability type.

**Methods:**

We surveyed business owners and decision-makers at companies with fewer than 100 employees resulting in a sample of 393 company respondents. Through descriptive analyses, we examined variations in respondents’ willingness to hire and the prevailing attitudes among the company leaders sampled. We explored how employer attitudes can either hinder or support the hiring of people with disabilities. We conducted multivariate analysis to explore the connections among attitudinal barriers, facilitators, and willingness to hire individuals with various disabilities, reflecting disability’s heterogeneous nature.

**Results:**

Our findings reveal that, in terms of hiring people with disabilities, the most important concerns among employers are: inability to discipline, being unfamiliar with how to hire and accommodate, and uncertainty over accommodation costs. These concerns do not differ between employers covered by the Americans with Disabilities Act (ADA) and non-covered employers. However, ADA-coverage may make a difference as ADA-covered employers are more likely to say they would hire an applicant with a disability. We find that for small companies (less than 15 employees), the positive effect of the facilitators (positive perceptions about workers with disabilities) almost completely offsets the negative effect of the barriers. However, for the larger companies, the marginal effect for an additional barrier is significantly more predictive than for an additional facilitator. Among the disabilities we examined, employers are least likely to hire someone with blindness, followed by mental health disabilities, intellectual disabilities, deafness, and physical disabilities, underscoring that employers do not view all types of disabilities as equally desirable at work.

**Conclusions:**

Understanding small employers’ underlying concerns and effectively addressing those factors is crucial for developing effective intervention strategies to encourage small employers to hire and retain people with different disabilities. Our results suggest greater openness among ADA-covered employers to hiring people with disabilities, but the perceived barriers indicate a need for ongoing information on effective intervention strategies to increase disability hiring among all small employers.

**Supplementary Information:**

The online version contains supplementary material available at 10.1007/s10926-024-10201-2.

## Introduction

In 2023, the employment rate of working-age people with disabilities in the United States was 37.3%, compared with 75.5% of persons without disabilities, despite evidence that, relative to their non-disabled counterparts, non-employed people with disabilities are as likely to want a job but less likely to be actively searching [1]. Even when they are actively searching, people with disabilities are twice as likely as others to be unable to find work [2]. Despite employment-related legislation, policies, and programs that attempt to close this gap, individuals with disabilities continue to face significant barriers in accessing employment opportunities [3, 4]. Their un- and underemployment stems from both “supply side” (those seeking jobs) and “demand side” (employer) factors [5, 6]. Because employers make the decisions to hire people with disabilities, this study focuses on the latter. We explore employers’ stereotypes and attitudes that act as barriers or facilitators to hiring people with disabilities. Furthermore, rather than treating disability as a homogeneous group, this study investigates variations in the willingness of small businesses to hire individuals with different types of disabilities to help determine the effect those differences may have.

We focus on small businesses with fewer than 100 employees because they account for 97% of businesses in the U.S., employ over half the workforce [7], and are considered “the lifeblood of the U.S. economy” [8]. Even though companies with over 15 employees are subject to the workplace non-discrimination requirements of the Americans with Disabilities Act (ADA) [9], extant research indicates that only 12% of small companies (5–14 employees) and 29% of medium sized companies (15–249 employees) employ people with disabilities, compared with 68% of large employers [10]. Nevertheless, most studies on increasing disability employment focus on concerns and practices that are relevant and appropriate for larger businesses. They do not adequately account for the differences in capacity, priorities, and operational procedures between large and small companies [11]. Various studies show that the *size of the organization and industry* are correlated with hiring intentions and/or disability hiring. Larger organizations are more likely to hire people with disabilities [10, 12]. Given the limited knowledge about hiring among small businesses, as well as early indicators that these types of organizations are less likely than large ones to hire people with disabilities, research is needed to further explore the unique barriers and facilitators facing small businesses.

Most of the relevant research on employment and employer hiring intentions studies people with disabilities as a homogenous and monolithic group [13, 14] or chooses to study a single type of disability [15, 16]. Aligned with prior arguments that “observers automatically categorize individuals according to several disability subtypes” [17] p. 356], we expect that there will be substantial differences in employers’ willingness and intention to hire people with disabilities based on the type of disability. This is likely due to differences in the extent and level of the employers’ perceived stereotypes and attitudinal biases towards people with different disabilities [17]. The type of disability and employers’ perceived biases concerning job performance of those employees can also affect an employer’s decision to hire people with certain disabilities [18].

Multiple studies corroborate that employers are more reluctant to hire people with any type of disability, indicating the presence of discrimination [19, 20]. Discrimination also appears to be a factor influencing hiring decisions when specific types of disability are considered. Studies report that employers voiced concerns about certain types of disabilities, for example, *people who use wheelchairs *[21, 22]*, people with visual impairments *[23, 24], *people with psychiatric disabilities *[25] and *people with developmental disabilities* [26]**.** Similarly, recent data on employment rates of people with disabilities show that individuals with hearing difficulties have the highest employment rate at 52%, closely followed by vision (40.3%), cognitive (31.9%), and ambulatory (23.1%) disabilities [27]. However, the majority of these studies focus on a single type of disability. Given these findings, there is value in exploring employer attitudes toward different disability types in a single, comparative study.

This study explores **three research questions** designed to inform tailored strategies that foster a more inclusive and diverse workforce: First, how do small businesses perceive and prioritize various barriers and facilitators when considering the employment of individuals with disabilities and what is the moderating role of the Americans with Disabilities Act (ADA)? Second, to what extent do small businesses exhibit favorable perceptions towards certain types of disabilities over others when making hiring decisions? Third, what is the relationship between barriers and facilitators and stated hiring likelihood?

In this study, we focus on barriers and facilitators identified in the literature. Importantly, many of these barriers are based on stereotypes of people with disabilities, such as the stereotype of incompetence leading to the barrier of employers believing candidates with disabilities are not qualified, or lack of knowledge, such as uncertainty about accommodations leading to the barrier of employers worrying about the cost to accommodate. In contrast, facilitators come largely from contextual aspects, such as the legal or competitive environment or the organizational culture, all of which can serve to encourage disability hiring.

### Stereotypes and Barriers

The literature reports several prominent employer-related attitudinal barriers to hiring people with disabilities in general. First, numerous scholars cited *stigma* (or perceived negative attitudes among some employers) as a key barrier to employers’ hiring intentions and/or actual hiring of people with disabilities [12, 23, 25, 26, 28, 29]. Some research has reported specific negative beliefs or stereotypes that employers hold concerning the abilities, capabilities, productivity, and potential impact on coworkers and customers, of employees with disabilities [3, 24, 30]. For example, some stereotypes about people with disabilities may be that they are helpless, unsociable, dependent, unhappy, or less competent [31].

Second, several studies found that *lack of knowledge and experience* with disability, and how to onboard, support, manage, and retain employees with disabilities, hinders hiring intentions and/or hiring of people with disabilities [3, 12, 24, 30], particularly in small businesses that may not have human resource departments and may take an informal approach to human resource management [32]. Lack of knowledge and experience with disability can increase the use of stereotypes, which can heighten concerns relating to the qualifications, recruitment, and selection process of applicants with disabilities, integrating people with disabilities in the organization, occupational and health concerns, and disciplinary action and termination of employment of an employee with a disability [3].

A third major barrier to hiring intentions and/or hiring of people with disabilities relates to *the cost of accommodations*, both real or perceived, by the employer [3, 26, 28, 30]. Despite evidence that the costs of accommodations are in most instances minimal, many small employers still fear that accommodating an employee with a disability will be costly [5, 33]. In addition to concerns about potential structural accommodation costs, it is worth noting that research has shown that employers worry about potential *“administrative burden”* regarding the extra time and effort that supervisors may need to commit to manage the work of employees with disabilities [30].

### Facilitators

The literature also reports an array of demand-side promotors (or facilitators) to employers’ intentions to hire and/or hiring of people with disabilities. For example, *disability employment legislation and policies* were shown to serve as facilitators [12, 21, 24]. In addition, *previous work experience with employees with disabilities *[12] and *need to gain a competitive advantage *[30] were also shown to promote hiring and hiring intentions. Finally, *organizational climate, culture, and policies that are committed to diversity and inclusion *[12, 25, 34] and the hiring organization’s *pro-social inclinations and a sense of social responsibility* were found to promote hiring intentions and/or hiring of people with disabilities [30]. In addition to promoting hiring, characteristics such as *workplace culture, co-worker support, and the presence of an effective diversity, equity, and inclusion (DEI) policy* increases the engagement of workers with disabilities in the company as evidenced through heightened organizational citizenship behavior [35].

## Survey Development and Procedure

A team of researchers from the Burton Blatt Institute at Syracuse University and Rutgers University, including the co-authors of this study, developed a set of questions based on a thorough review of extant qualitative and quantitative studies identifying demand side barriers and facilitators to disability employment, particularly focused upon the experiences of small businesses.

The questions were fielded as part of the on-line SSRS Multi-client Small Business Omnibus Survey conducted in August 2022. The disability-related questions were preceded by unrelated questions developed by another SSRS client and prefaced with the following statement: “Now, changing the subject… An individual with a disability is defined as a person who has a physical, sensory, cognitive, or mental impairment that substantially limits one or more major life activities.”

SSRS established targets for interviewing based on (1) number of employees, (2) type of business, and (3) region. The targets for number of employees were a stratification of the sample across four employee range groups.

Data were weighted to be representative of the target population and calibrated to correct for the sample target and differential nonresponse along Industry Type, Number of Employees, and Census Region. The distributions for the parameters were obtained from the Dun & Bradstreet (D&B) database. Among companies with fewer than 100 employees in the US, 62% have fewer than five employees and only 9% have 20 or more employees [7]. However, the survey stratifies across size, as a result, 31.6% of survey respondents have fewer than five employees and 34.8% have 20 or more employees. Because of the composition of the sample compared to the true size distribution of small companies in the US, companies with 20 or more employees had significantly lower weights than the smaller companies.

A total of 500 business owners, presidents, and general managers of small businesses with 100 or fewer employees completed the online survey. Respondents were required to identify themselves as the person most or partially responsible for making the day-to-day decisions in the company (69.2% were owners and 30.2% were managers of the business operation).

For the data analysis, we excluded responses from 93 businesses with only one employee and 14 businesses who responded “not sure” or “prefer not to answer” on all questions related to barriers and facilitators. Although “not sure” is considered a valid response in the analysis, replying “not sure” to *all* questions indicated the respondent was not engaged with the survey. The characteristics of these 14 businesses did not differ significantly from the rest of the sample. This reduced the sample down to 393 organizations with more than one employee. Respondents in the final sample work in 41 different states, with 21.9% of them operating in home-based businesses (*n* = 86). Over half (56.7%) had 15 or more employees, meaning that they are covered under the employment provisions of the ADA.

Because the original sample had an underrepresentation of small companies compared to the actual composition of small businesses in the U.S. and the sample is weighted to address this imbalance, the weighted characteristics differ from the sample in several notable ways. First, a much larger proportion of the weighted sample have fewer than 15 employees (i.e., not covered by the ADA) than the unweighted sample. Additionally, the weighted sample represents a higher proportion of companies with zero employees with disabilities. These differences caused by the weighting are mitigated by dividing the sample into ADA and non-ADA-covered companies.

## Measures

### Outcome Measure

#### Likelihood to Hire

Participants were asked “If you were to hire one or more persons with a disability into your business, how likely might you be to hire persons with the following categories of disabilities?” Participants were asked to rate their response for each disability type on a 3-point scale of 1 = not likely, 2 = a little likely, and 3 = very likely, with options for not sure and prefer not to answer. The categories of disability types were intellectual and developmental disabilities (IDD), blindness, deafness, physical or mobility disabilities, and mental health disabilities (referred to on the survey as mental illnesses). These categories were randomized during the administration of the survey. For parts of the analysis, the likelihood variables were recoded into a binary variable where 1 is “very likely” and 0 is all other responses (not likely, a little likely, and not sure). We coded responses of not likely, a little likely, and unsure together as they all convey hesitation in hiring people with disabilities whereas very likely does not.

### Independent Variables

#### Barriers

Barriers to hiring people with disabilities were captured in a question that provided participants with a list of potential concerns and asked them to rate each based on their level of concern using a 3-point scale where 1 = not a concern, 2 = somewhat of a concern, and 3 = major concern, with options for not sure and prefer not to answer. Barriers listed were: (1) People with disabilities do not apply to the jobs; (2) They apply but do not meet the qualifications; (3) That the respondent is not familiar with how to hire or accommodate an employee with a disability; (4) That employees with disabilities are not as productive; (5) That you cannot discipline or fire a worker with a disability due to possible legal issues; (6) Uncertainty about cost of accommodations; (7) Concerns that other employees may feel it is unfair that an employee with a disability receives an accommodation; (8) Belief that overall costs outweigh the benefits; and (9) Concerns about reactions of clients or customers when interacting with employees with disabilities. These potential barriers were informed by the literature identifying both practical challenges to hiring people with disabilities (such as people with disabilities do not apply to the job), uncertainty on the part of the employer (such as not familiar with how to hire and accommodate), and common negative stereotypes about people with disabilities, their capabilities, and difficulties related to employing them. The statements were randomized during the administration of the survey to reduce any response bias that may have arisen based on the order of the questions. For parts of the analysis, barriers were recoded as a binary variable. In this recoding, 0 represents “not a concern,” while 1 represents “at least some concern” and includes the original responses of “somewhat a concern,” “major concern,” and “not sure.”

#### Facilitators

Facilitators to hiring people with disabilities were captured in a question that provided participants with a list of factors to consider when hiring employees and asked them to rate each of them based on how important the factor was in their decision to hire or not hire, on a 3-point scale where 1 = not important, 2 = somewhat important, and 3 = very important, with options for not sure and prefer not to answer. Facilitators listed were: (1) Factors or personal experience or familiarity with disabilities motivating the respondent to hire; (2) Hiring people with disabilities improves the company’s business image in the community; (3) Employees with disabilities contribute to a positive and more productive work environment; (4) Employees with disabilities contribute to business’s bottom-line through knowledge and productivity; (5) The business has a diversity hiring policy that includes recruiting and hiring qualified people with disabilities; (6) Respondent is motivated to hire people with disabilities after hearing or reading about costs vs. benefits of hiring them; and (7) The respondent was motivated by government policies such as those encouraging federal contractors and employers to hire people with disabilities. These statements were also randomized during the administration of the survey. For parts of the analysis, facilitators were recoded as a binary variable were 0 represents “not important” while 1 is “at least somewhat important” and includes original responses of somewhat important, very important, or not sure.

### Moderating Variables

#### Covered by the Americans with Disabilities Act (ADA)

Title I of the ADA prohibits companies with 15 or more employees from discriminating against qualified individuals with disabilities and requires these employers to provide reasonable accommodations to qualified candidates and employees with disabilities. Based on the number of employees reported, we constructed a binary variable where 0 = not covered by the ADA (fewer than 15 employees) and 1 = covered by the ADA (15 or more employees). Among the respondents used in the analysis, 56.7% were covered by the ADA accounting for 8% of the weighted sample (Table 1).

**Table 1:** Sample characteristics unweighted, weighted and by company Size

	Unweighted % of total	Weighted % of total	Weighted % non-ADA	Weighted % ADA	p value: weighted ADA v non-ADA
Number of employees					
2–14	43.3	92.0			
15–99	56.7	8.0			
Respondent role					0.00
Owner	63.0	80.0	82.0	54.0	
Manager	37.0	20.0	18.0	46.0	
Number of years in business					0.07
0–4	13.6	18.4	19.0	10.9	
1–9	16.9	16.4	16.4	16.8	
10–19	24.3	28.7	29.2	23.3	
20 or more	45.3	36.5	35.4	49.0	
Annual revenue					0.00
Under $100 K	8.7	20.4	21.9	2.7	
$100 K to < $500 K	20.7	34.9	36.7	15.2	
$500 K to < $1 M	19.9	20.9	21.6	13.5	
$1 M to < $5 M	35.7	20.3	17.7	49.8	
$5 M or more	15.0	3.5	2.1	18.9	
Industry					0.42
Agriculture, forestry, or mining	3.1	5.2	5.4	2.2	
Construction	13.5	11.5	11.4	12.4	
Manufacturing	9.4	5.0	4.6	9.2	
Transportation, communication, or utilities	6.1	6.4	6.1	10.0	
Wholesale trade	1.8	3.4	3.4	2.8	
Retail trade	15.5	15.0	14.8	16.3	
Finance, insurance, or real estate	10.2	9.8	10.1	6.4	
% with disabilities					0.00
0%	77.9	87.1	88.2	72.6	
0–2.99%	4.4	0.5	0.1	5.7	
3–6.99%	6.8	0.9	0.0	12.3	
7% or more	10.9	11.6	11.7	9.3	
Disability relationship					
Self (including veteran with a disability)	6.6	7.7	7.9	4.5	0.20
Family member	16.5	19.0	19.0	18.9	0.98
Relative or close friend	28.0	24.6	23.7	34.2	0.06
Co-worker	12.7	6.6	5.7	16.2	0.01
None of these	46.1	50.1	50.9	41.8	0.13

*N* = 393

### Control Variables

#### Respondent Job Category

Respondents were asked to report their job category (i.e., whether they were an owner or a manager). We included this variable because, although it is not highlighted in the literature, we expected that managers may feel more constrained by company policies, protocols, and compliance issues, potentially making them more circumspect in their hiring practices. Whereas, business owners, especially those running smaller businesses, might have more flexibility in decision-making, which could allow them to adopt more inclusive attitudes and practices. Owners represent almost two-thirds (63%) of our overall sample. Respondents from small companies (fewer than 15 employees) were more likely to be owners than respondents from larger companies. (Table 1).

#### Employees with Disabilities

This variable was calculated based on the reported number of employees and the number known to have a disability. For the multivariate analysis, we collapsed this into a binary variable where 1 indicated the company had at least one employee with a disability. 77.9% of respondents reported they had no employees with disabilities and 22.1% reported having employees with disabilities (Table 1).

#### Number of Years in Business

We include this in the multivariate models as a continuous variable. 45.3% of the sample have been in business for 20 or more years.

#### Industry

Respondent identified their industry as Agriculture, Forestry or Mining Construction; Manufacturing; Transportation, Communication, or Utilities; Wholesale Trade; Retail Trade; Finance, Insurance, or Real Estate, or Business or Personal Services. Because of the small number of responses from some categories, for the multivariate analysis we combined agriculture, construction, manufacturing, and wholesale trade into one category of “other.”

#### Knowing Someone with a Disability

Respondents were asked to select all from a list in response to “Do you or does someone you know have a disability?” The valid response options included: I have a disability; I am a Veteran with a disability; I have an immediate family member with a disability; I have a relative or close friend with a disability; I have coworker(s) with a disability; None of these apply. Respondents could choose all that apply. Most, but not all, veterans with a disability also chose “I have a disability.” For analysis, we collapsed the categories into a binary variable where 1 indicated that the respondent had a disability or knew at least one person with a disability from the categories mentioned. Around 7% of the organizations were disability owned (see Table 1). 53.9% of the sample indicated they had some personal relationship with disability.

## Data Analyses

To assess how small businesses perceive and prioritize various barriers and facilitators when considering the employment of individuals with disabilities, we computed descriptive statistics for each of the barriers and facilitators for the two groups (ADA and non-ADA). To identify differences based on the size of the company (and thus whether the company is covered under the ADA), we computed Pearson *χ*^2^ to assess the difference between the size groups in the nominal variables and a *t*-test to identify differences in the continuous variables.

To assess the extent to which small businesses exhibit favorable perceptions towards certain types of disabilities when making hiring decisions, we computed descriptive statistics for the likelihood of small businesses to hire each type of disability.

We then looked at the relationship between the barriers and facilitators and the likelihood to hire each disability type. A correlation table indicated that important controls to include are the type of respondent, years in business, number of employees, percent of employees with a disability, and whether the respondent lacked a connection to any person with a disability (self, family member, relative or close friend, or coworker).

We conducted two sets of multivariate analyses. We conducted a set of linear regressions where the dependent variable is the composite hiring variable. In the first model, the independent variables include the composite barriers and facilitators. In the second model, the independent variables include the full set of 10 barriers and 7 facilitators coded as binary variables.

To better understand how barriers and facilitators differ based on the type of disability, we used the same two sets of independent variables to estimate separate logit models for each disability type where the dependent variables are binary variables indicating if the employer is likely to hire a particular type of disability.

We conducted all analyses for the ADA and non-ADA businesses. This approach serves two purposes. First, it allows us to identify differences by size. Second, in the absence of dividing the sample, the views and perspectives of the larger companies are not well represented in the results because of the weighting scheme. However, while there are fewer of these larger companies, they are important to consider because they have more employees and thus more opportunities for disability hiring.

## Results

**Question 1: How do small businesses perceive and prioritize various barriers and facilitators when considering the employment of individuals with disabilities and what is the moderating role of the Americans with Disabilities Act (ADA)?**

### Barriers

Given a list of potential barriers to hiring employees with disabilities, respondents were asked to rate each based on a scale of not a concern, somewhat a concern, major concern and unsure. The three areas that created the most concern were: (1) cannot discipline; (2) unfamiliarity with how to hire and accommodate an employee with a disability; and (3) uncertainty about the cost of accommodation. Fairness for other employees and client/customer reaction were the least concerning (See Table 2). Notably, for each of the statements, a relatively low percentage of respondents expressed any level of concern (this may be a case of social-desirability bias, a known challenge in disability research) [36].

**Table 2:** Attitudinal barriers to hiring people with disabilities

	Overall % with concerns	Non-ADA % with concerns	ADA % with concerns	*p*-value for difference
Cannot discipline	38.5%	39.0%	33.2%	0.32
Unfamiliar with how to hire and accommodate	36.5%	36.9%	32.1%	0.40
Uncertainty about cost of accommodation	35.1%	35.5%	31.6%	0.49
People with disabilities did not apply to the job(s)	27.8%	27.4%	32.3%	0.38
Potential cost outweigh benefits	26.6%	26.7%	26.2%	0.93
Not as productive	25.4%	25.1%	28.5%	0.51
Not qualified	24.8%	24.0%	31.8%	0.15
Fairness for other employees	20.6%	20.1%	27.2%	0.16
Client/customer reaction	13.0%	12.3%	22.1%	0.02
Overall barriers	1.30	1.29	1.37	0.15

% with Concerns includes Somewhat a concern, Major Concern, and Don’t Know. *p*-value based on adjusted Wald test for each binary variable

For the questions that aligned with other studies, the magnitude of the concerns found in this study were in the same range, albeit slightly lower for some concerns. For example, 38.5% of this sample said “Cannot discipline or fire a worker with a disability due to possible legal issues” was at least somewhat a concern. In a similarly worded question, Gasper et al. found 50.4% of companies with 5–49 employees and 43.6% of those with 50–249 employees said it was any concern. Both studies found fewer than 30% of respondents had concerns about attitudes of customers and co-workers [10]. This contributes to the external validity of the results and likely generalizabilty outside of this study’s sample.

A comparison of attitudinal barriers between ADA and non-ADA companies found few significant differences in the prevalence of the concerns. However, the ranking of the concerns varied by employer size (Table 2). Inability to discipline an employee for fear of legal action was the top attitudinal barrier for companies regardless of their size and whether they are covered under the ADA. This seems counterintuitive since companies with fewer than 15 employees are not covered by the law that could potentially impose a penalty. In a supplementary analysis, we considered whether non-ADA-covered companies were more likely to share this concern if they operated in states with disability anti-discrimination laws that cover companies with lower employee thresholds than the one set by the ADA. However, we found that companies with fewer than 15 employees were equally likely to share the concern, regardless of state law. Small companies also tended to rank cost-related barriers (uncertainty about job accommodations and costs outweigh benefits) as bigger concerns, whereas large companies were more concerned about people with disabilities not applying to jobs and not being qualified (Table 2). Given the smaller budgets and resources typically available to smaller organizations, it is not surprising that those companies would be more concerned with costs than larger employers.

Finally, we created a measure of overall concerns as the mean of non-missing values of the nine concerns to conduct comparisons by company size. Our results show no statistical difference (*p*-value = 0.15) in the overall concerns between ADA-covered and non-ADA-covered companies (Table 2). This indicates that being covered by the ADA was not a significant predictor of an employer’s level of concern regarding potential barriers to hiring people with disabilities. Increased resources may result in larger companies being less concerned with cost barriers when considering hiring people with disabilities, but other potential barriers still serve as significant concerns for those employers.

### Facilitators

Like the attitudinal barriers, respondents seemed to consider the facilitators as separate issues. Only 25.7% of the sample gave the same response to all questions. When asked about attitudes that may motivate companies to employ people with disabilities, respondents reported the belief that employees with disabilities contribute to the ‘bottom line’ through their knowledge, skills, and productivity and that hiring people with disabilities contributes to a positive and more productive work environment as the two most important facilitators (see Table 3). The two least important factors were government policies, such as those that encourage federal contractors and employers to hire people with disabilities and hearing or reading about the positive costs versus benefits of hiring this population (Table 3). Overall, employer size affected the reported importance of the facilitator variables, but it did not affect the order of importance (Table 3). For example, while both ADA-covered and non-ADA-covered employers rated government policies as one of the least important factors, ADA-covered employers were much more likely to say government policies were a very important factor in their decision, likely because only these larger companies are affected by these government policies.

**Table 3:** Attitudinal facilitators to hiring people with disabilities

	Overall % Important	Non-ADA % Important	ADA % Important	p-value
Positive work environment	73.8%	73.1%	81.8%	0.09
Bottom line	69.7%	69.0%	77.5%	0.13
Company policy	60.6%	59.4%	74.9%	0.01
Improves corporate image	59.0%	58.0%	70.7%	0.03
Personal familiarity	53.9%	52.5%	69.1%	0.01
Costs versus benefits	46.2%	44.9%	61.2%	0.01
Government policies	40.0%	38.4%	57.8%	0.00
Overall facilitators	1.79	1.78	2.03	0.00

% Important includes somewhat important, very important and don’t know. *p*-value based on adjusted Wald test of each binary variable

Finally, we calculated overall differences by company size. Based on a composite measure of facilitators defined as the mean of non-missing values of the seven facilitators, ADA-covered companies displayed a higher overall level of facilitators compared with smaller companies (Table 3).

**Question 2: To what extent do small businesses exhibit favorable perceptions towards certain types of disabilities over others when making hiring decisions?**

When asked about likelihood to hire a person with a specific type of disability, the results indicate that respondents assessed each disability type separately. Only 20% of respondents gave the same response for each of the five disability types, indicating that they differentiated between disability types rather than viewing all people with disabilities as the same.

**Fig. 1:**
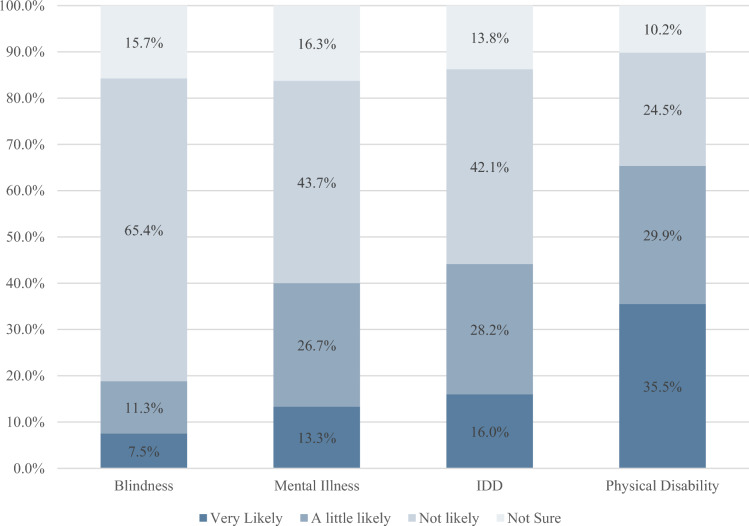
Likelihood of hiring people with different categories of disability types

Respondents said they were least likely to hire people who are blind (only 7.5% responded “very likely” and 65.4% responded not likely) and most likely to hire people with a physical disability (35.5% very likely and 10.2% not likely) (Fig. 1).

We computed the likelihood of hiring people with different types of disabilities separately for each of the ADA groups. Although the percentages differed somewhat, the order of willingness to hire remained from least to most likely: Blindness, mental health disabilities, IDD, deafness, and physical disabilities. ADA-covered companies were more likely to say they were “very likely” to hire people with each type of disability. The difference is statistically significant for blindness, mental health disabilities, and IDD (Table 4).

**Table 4:** "Very likely" to hire, by disability type and employer size

	Non-ADA	ADA	*P*-value
Blindness	6.9%	15.4%	**0.03**
Mental health disabilities	12.3%	25.3%	**0.01**
IDD	15.2%	25.7%	**0.04**
Deafness	24.9%	32.1%	0.20
Physical disability	35.0%	41.9%	0.25
Overall willingness to hire	1.90	2.14	**0.00**

*P*-value based on Pearson Chi Square adjusted for weighting scheme. Significant values (< 0.1) highlighted in bold. Overall willingness to hire score consists of the mean of non-missing values for 1–3 (very likely, a little likely, not likely). Not sure is considered missing

We computed a composite variable as the mean of non-missing values and tested the relationship between ADA status and this overall likelihood to hire a person with a disability. ADA-covered employers were significantly more likely to say they would hire an applicant with a disability with a mean value of 2.14 compared with 1.90 for the smaller employers.

**Question 3: What is the relationship between barriers and facilitators and hiring likelihood?**

Next, using the standard controls explained earlier, we conducted four sets of analyses where we examined both composite scores and individual barriers/facilitators to understand their impact on the overall likelihood to hire individuals with disabilities, as well as the likelihood to hire each specific type of disability.

In Model 1 (Table 5), which looks at the relationship between the overall likelihood to hire and the composite scores for barriers and facilitators, we find that for small companies (less than 15 employees), the positive marginal effect of the facilitators almost completely offsets the marginal negative effect of the barriers. However, for larger companies, the marginal effect of the barriers exceeds the marginal effect of the facilitators. Industry type was also shown to matter for the ADA-covered employers, with transportation having significantly lower hiring likelihood than most other industries. Tying intentions to actual hiring, these results match with the Bureau of Labor Statistics’ data on employment of people with disabilities in 2023, which found that more people with disabilities were employed in retail trade (13.0% of employed people with disabilities) than in transportation and utilities (6.1%) [37].

**Table 5:** Model 1-OLS regression predicting overall likelihood to hire from latent constructs of concerns and facilitators by company size subpopulations

	Model 2a Non-ADA	Model 2b ADA
Owner (versus manager)	− 0.109	− 0.064
Percent disability employment	− 0.026	0.025
Years in business	− 0.003*	− 0.003
Have disability relationships	0.080	0.122*
Industry		
Retail	0.073	0.227**
Finance	0.062	0.199
Business	0.099	0.177*
Other	0.052	0.185*
Avg barriers	− 0.227*	− 0.299***
Avg facilitators	0.188**	0.112
Constant	0.259***	0.188
*R*-Squared	.1785	.1796

*Weighted N* = 156–379. Model 2a uses the non-ADA subpopulation while Model 2b uses the ADA subpopulation. The omitted industry type is Transportation. ***p < 0.01, **p < 0.05, *p < 0.1

Knowing someone with a disability is associated with an increase in the overall likelihood of being willing to hire a person with a disability for ADA-covered businesses by 0.122. However, having a connection to someone with a disability was not a significant influence on hiring likelihood for smaller businesses.

In Model 2 (Table 6), we evaluated the effect of the overall measures of barriers and facilitators on being “very likely to hire” each type of disability and found that, for large companies, barriers are negatively associated with likelihood to hire three of the five types of disabilities while the role of facilitators is not statistically significant (Table 6).

**Table 6:** Model 2-Odds ratio logit of barriers and facilitators of hiring each type of disability by company size subpopulations

	Non-ADA		ADA	
	Avg barrier	Avg facilitator	Avg barrier	Avg facilitator
IDD	0.134	7.725	0.277	1.618
Blindness	0.0704	5.055	0.154	1.567
Deafness	0.269	3.997	0.206*	2.573
Physical	0.252	3.634	0.059***	2.042
Mental health disabilities	0.176	1.882	0.166*	2.240

*N* = (156–379) Independent variables include all standard controls and standardized latent constructs of Concerns and Facilitators. Standard controls not shown.***p < 0.01, **p < 0.05, *p < 0.1

Next, we explored each of the barriers and facilitators separately, rather than the overall constructs, to identify specific attitudes that may affect the likelihood of hiring people with disabilities.

In Model 3 (Table 7), we consider the role of each barrier and facilitator in predicting the overall likelihood of hiring a person with a disability. For small businesses, concerns about productivity affect the likelihood of hiring, whereas for large businesses, the perception that they cannot discipline an employee with a disability discourages hiring. For large businesses, personal familiarity significantly increases the likelihood of hiring people with disabilities. For both ADA and non-ADA businesses, newer businesses indicated more hesitancy to hire people with disabilities.

**Table 7:** Model 3-OLS regression predicting overall likelihood to hire using individual concerns and facilitators, by company size subpopulations

	Non-ADA	ADA
Owner (versus manager)	− 0.242	− 0.143
Pct disability employment	0.368	0.910**
Years in business	− 0.007**	− 0.00672*
No disability relationships	− 0.182	− 0.191*
Industry		
Retail	0.043	0.307
Finance	0.036	0.158
Business	0.085	0.178
Other	− 0.094	0.167
Did not apply	0.127	0.038
Not qualified	− 0.037	0.208*
Not familiar with how to	− 0.061	− 0.062
Not as productive	− 0.236*	− 0.075
Cannot discipline	− 0.077	− 0.312**
Uncertain about cost of accommodation	− 0.016	− 0.161
Fairness for other employees	0.293**	0.043
Cost outweigh benefits	− 0.096	− 0.118
Client/customer reaction	0.132	− 0.048
Personal familiarity	0.122	0.367**
Improves corporate image	0.004	− 0.082
Positive work environment	0.097	− 0.068
Bottom line	0.089	0.076
Company policy	0.111	− 0.223
Costs versus benefits	0.044	0.114
Government policies	0.020	− 0.011
Constant	2.044***	2.296***

In Model 4 (Appendix), we ran separate models, one for each disability type, including the standard controls and all nine barrier items and seven facilitator items.

These results suggest that barriers and facilitators may be differentially important depending on the size of the company and type of disability the candidate has (see Tables A1 and A2 in the Appendix). Unfortunately, the results do not point to a discernable pattern. To account for the possibility of spurious results due to the analysis of numerous related independent variables, we conducted robustness tests. These tests involved estimating the model using different specifications of the independent variable including a multinomial logit using the four response levels (not likely, a little likely, very likely, and not sure) and regressions excluding “not sure” and considering the other values as continuous.

Our results included in the appendix are similar to our findings from Model 3 in Table 7. Our findings in Model 3 showed that small companies are particularly concerned about productivity. When breaking this down by disability type, we found that this concern was a significant predictor of hiring likelihood for deafness and physical disabilities, but not for other disabilities. In addition, in Model 3, we found concern about the inability of larger employers to discipline employees with disabilities hampered their hiring intentions, but that personal familiarity increased hiring intentions. In Model 4, we find that the concern about discipline is most significant when considering people who are blind or deaf and the personal familiarity is most significant in increasing hiring of people who are blind.

## Discussion

This study identified key insights related to the concerns and facilitators that affect disability hiring. The most significant concerns identified by employers include the fear of not being able to discipline employees with disabilities, uncertainty about how to effectively hire and accommodate individuals with disabilities, and concerns regarding the potential costs of accommodations. These concerns were generally consistent across both ADA-covered (15–99 employees) and non-covered employers (fewer than 15 employees) in the sample, indicating a shared set of apprehensions regardless of size and legal obligations.

Despite the similarities in concerns, ADA-covered employers reported a higher prevalence of facilitators and expressed a greater willingness to hire individuals with disabilities compared to smaller employers. This suggests that while the concerns are shared, ADA-covered employers may be better equipped to address these challenges, potentially because they are responding to ADA requirements or because they are larger and have the resources and infrastructure to address the challenges.

Furthermore, our analysis revealed differences in the impact of barriers and facilitators on disability hiring between ADA-covered and non-covered employers. Attitudinal barriers and common stereotypes about people with disabilities (e.g., they are not as productive, are difficult to discipline, cost a lot to accommodate, or clients/customers will react negatively) are more powerful predictors of willingness to hire in companies covered by the ADA (15 or more employees) than in smaller companies. For non-ADA employers, the marginal effects of barriers and facilitators were approximately equal, indicating a balanced influence of these factors on their hiring decisions. In contrast, the marginal effect of an extra barrier is more important than an extra facilitator for ADA-covered employers suggesting that small businesses with more than 15 employees may be more responsive to interventions and information to reduce attitudinal barriers, as opposed to interventions that reiterate and promote attitudinal facilitators.

In terms of specific barriers and facilitators, we found that concerns about productivity reduce hiring intentions for smaller businesses. For larger businesses, the perception that they cannot discipline an employee with a disability discourages hiring. In large businesses, personal familiarity significantly increases the likelihood of hiring people with disabilities. For both ADA and non-ADA businesses, newer businesses indicated more hesitancy to hire people with disabilities.

Additionally, we find employers do not view people with disabilities as a monolithic group. The survey asked the question: “if you were going to hire a person with a disability, would you hire someone with this type of disability?” The question put respondents into the mindset that they had a position open and were hiring a person with a disability, so differences seen across disability types are based on beliefs that not all types of disabilities are equally desirable in the workplace. Our analysis showed that employers were least likely to hire someone with blindness followed by mental health disabilities, intellectual disabilities, deafness, and physical disabilities. This finding adds to the literature on employer attitudes, which typically operationalizes disability as a monolithic group or focuses solely on one type of disability, thereby overlooking the inherent variability within the category [13–16].

These findings underscore the complex landscape of disability hiring, highlighting both shared concerns and divergent approaches between ADA-covered and non-ADA-covered employers. Understanding these nuances is essential for developing targeted strategies to promote disability inclusion in the workplace.

Our research has clear implications for employers, job placement specialists, disability advocacy organizations, training organizations, policy makers, and researchers. Employers need to recognize the different subtle or implicit biases that may be affecting their hiring decisions with an understanding that these biases may differ based on the prospective employee’s type of disability. Once they have established awareness, organizations can develop tailored strategies, including educating human resource managers and staff, to dispel misconceptions related to specific types of disabilities. Understanding employer attitudes can help vocational counselors, and job placement specialists, advocacy organizations, and organizations that provide disability training and support to provide tailored guidance and resources to both job seekers and prospective employers. Training organizations should show positive examples in the workplace by type of disability and create different interventions for businesses covered by the ADA and those that are not covered. It is also important for trainers to note that businesses with more than 15 employees may be more responsive to barrier-busting type interventions, as opposed to facilitator-promoting ones.

Groups seeking to increase employment for people with disabilities should consider what jobs match the preferences of jobseekers with disabilities and account for the size of the company. If they are targeting small businesses (fewer than 15 employments), they might focus on educating employers on how to accommodate and manage employees with disabilities (especially deaf employees or those mental health disabilities) while also addressing concerns related to discipline and productivity. If the employers being targeted are larger organizations (16–99 employees), a deeper focus on alleviating concerns regarding disciplining employees with disabilities (especially blind and deaf workers) while also addressing the productivity potential (with a focus on IDD) may produce the best return-on-investment.

Small businesses may need more interventions focused on addressing cost-related concerns, such as increased awareness around the real costs associated with most common workplace accommodations. On the other hand, larger organizations may benefit more from learning where and how to recruit to ensure qualified candidates with disabilities are applying to address their candidate-related concerns. Larger organizations are also more likely to hire people with disabilities when they know someone with a disability, so advocacy groups could consider a buddy program that pairs people with disabilities with recruiters at organizations for targeted outreach.

Finally, the findings from this study underscore the multifaceted nature of disability discrimination in the workplace and its implications for both policy and practice, highlighting the need for policy makers to consider the disparate effects of government policies designed to advance employment outcomes across all types of disability. As a first step, the Equal Employment Opportunity Commission could collect more detailed data by type of disability to identify trends in disability employment discrimination.

**Limitations:** A primary limitation of our study is the potential for social desirability bias, as is often a risk in disability-focused research. While our questions attempted to direct respondents to think in such a way as to limit this bias (e.g., framing as ‘if you were to hire’ rather than ‘would you hire’), the risk remains a limitation of our results.

A second limitation is that our study assesses the intent to hire rather than actual hiring behavior. The literature points to an “intention-behavior gap” [41]. This gap affects the strength of the conclusions about actual hiring that can be drawn from a survey focused on hiring intention. We were limited by our research design and future studies could use field studies with experimental designs to measure actual hiring (e.g. a resume study accounting for different types of disabilities to measure labor market discrimination).

Although it is valuable to address underlying concerns and reiterate the attitudes that promote the hiring of people with disabilities in general, we need to acknowledge how these attitudes affect people with different types of disabilities. At the same time, we need to recognize that segmenting people with disabilities into different categories based on type of disability can be as problematic as grouping them into one category. On the one hand, our survey, which divides disabilities into distinct groups, highlighted an important phenomenon. That is, without even considering the characteristics or strengths of an individual applicant, employers are more hesitant to hire people with some types of disabilities than others. This simple question indicates the existence of different stereotypes and discrimination.

On the other hand, any categorization, whether it be disability as a whole, or different types of disabilities, is an oversimplification of a person’s identity. Disabilities are diverse, and diversity can exist even within broad disability categories, and people with the same disability label can have very different abilities and needs, as well as accommodation types. In addition, disabilities intersect with other aspects of a person’s identity, such as race, gender, sexual orientation, and socioeconomic status. Focusing solely on disability labels may ignore these important intersectional dimensions of a person’s life. Scholars have noted the limitations of the framing of disability as the most important or defining aspect of an individual’s identity overshadowing other aspects of their identity, such as race, gender, or sexual orientation (e.g., a “master status”) [13, 38–40]. Although this framing allows us to draw parallels to other forms of oppression and highlight similarities between the experiences of people with disabilities and those who face oppression based on race, gender, or sexual orientation, it fails to recognize the varied experiences people with disabilities face.

Although insights into the intentions of employers to hire people with disabilities are valuable for understanding hiring barriers, it is important to note that research has shown that even positive intentions to hire people with disabilities do not always lead to behavior change, namely, actual hiring of people with disabilities [30]. One study found that concrete indicators of the workplace diversity climate (formal disability hiring and disability training) were stronger predictors of hiring people with disabilities, rather than the intention to hire [42].

**Future research:** There is a substantial difference in the willingness or concerns of employers about hiring an applicant with a disability based on the type of disability. More research is needed to identify the specific attitudes that explain this intention-behavior gap.

We have generated data that indicate that business-size affects an employer’s decision to hire an applicant with a particular disability. Further research is needed that compares the attitudes and experiences of ADA and non-ADA-covered employers of different sizes. Concurrent with that exploration, more research is needed on how small employers address the challenges of seeking out and onboarding, retaining, and advancing people with disabilities in their companies, compared with the experiences of large employers.

Our research asked survey respondents about their willingness to hire people with five broad categories of disabilities. Additional research should consider attitudes toward people with chronic health conditions and other types of disabilities. Future research should also consider the impact of having a disability combined with other marginalized identities.

## Supplementary Information

Below is the link to the electronic supplementary material.

Supplementary file1 (DOCX 21 KB)

## References

[1] Ali M, Schur L, Blanck P. What types of jobs do people with disabilities want? J Occup Rehabil. 2011;21:199–210. 20924777 10.1007/s10926-010-9266-0

[2] Bureau of Labor Statistics. Persons with a disability: labor force characteristics-2023. Accessed: Feb. 29, 2024. [Online]. Available: https://www.bls.gov/news.release/disabl.nr0.htm

[3] Bonaccio S, Connelly CE, Gellatly IR, Jetha A, Martin Ginis KA. The participation of people with disabilities in the workplace across the employment cycle: employer concerns and research evidence. J Bus Psychol. 2020;35:135–58. 32269418 10.1007/s10869-018-9602-5 PMC7114957

[4] Zijlstra F, Mulders H. Disabilities at work. In: Oxford research encyclopedia of psychology, 2022.

[5] Blanck P, Frieden L. Disability law and policy. Goleta: Foundation Press; 2020.

[6] Blanck P, Hyseni F, Goodman N. Economic inclusion and empowerment of people with disabilities. In: Handbook of disability: critical thought and social change in a globalizing world. Berlin: Springer; 2023. p. 1–22.

[7] United States Census Bureau. 2019 SUSB Annual Data Tables by Establishment Industry. United States Census Bureau, Feb. 2022. Accessed: Aug. 27, 2023. [Online]. Available: https://www.census.gov/data/tables/2019/econ/susb/2019-susb-annual.html

[8] Office of Advocacy. Small businesses generate 44 percent of U.S. economic activity. Accessed: Sep. 15, 2023. [Online]. Available: https://advocacy.sba.gov/2019/01/30/small-businesses-generate-44-percent-of-u-s-economic-activity/#:~:text=WASHINGTON%2C%20D.C.%20%E2%80%93%20Small%20businesses%20are,percent%20of%20U.S.%20economic%20activity.

[9] The Americans with Disabilities Act, 1990.

[10] Gasper J, Palan M, Muz B. Survey of employer policies on the employment of people with disabilities. Rep Prep Chief Eval Off CEO Off Disabil Employ Policy ODEP, 2020.

[11] Sequeria JM, Weeks KP, Bell MP, Gibbs SR. Making the case for diversity as a strategic business tool in small firm survival and success. J Small Bus Strategy. 2018;28(3):31.

[12] Iwanaga K, Chan F, Ditchman N, Tansey TN. Assessing workplace culture and disability inclusion climate: a preliminary study. J Appl Rehabil Couns 2021.

[13] Bezyak J, Moser E, Iwanaga K, Wu J-R, Chen X, Chan F. Disability inclusion strategies: an exploratory study. J Vocat Rehabil. 2020;53(2):183–8.

[14] Chan F, Strauser D, Maher P, Lee E-J, Jones R, Johnson ET. Demand-side factors related to employment of people with disabilities: a survey of employers in the Midwest region of the United States. J Occup Rehabil. 2010;20:412–9. 20602153 10.1007/s10926-010-9252-6

[15] Baldwin ML, Choe C. Re-examining the models used to estimate disability-related wage discrimination. Appl Econ. 2014;46(12):1393–408.

[16] Richards J, Sang K. Trade unions as employment facilitators for disabled employees. Int J Hum Resour Manag. 2016;27(14):1642–61.

[17] Stone DL, Colella A. A model of factors affecting the treatment of disabled individuals in organizations. Acad Manage Rev. 1996;21(2):352–401.

[18] Premeaux SF. Impact of applicant disability on selection: the role of disability type, physical attractiveness, and proximity. J Bus Psychol. 2001;16:291–8.

[19] Ameri M, Schur L, Adya M, Bentley FS, McKay P, Kruse D. The disability employment puzzle: a field experiment on employer hiring behavior. ILR Rev. 2018;71(2):329–64.

[20] Bjørnshagen V, Ugreninov E. Disability disadvantage: experimental evidence of hiring discrimination against wheelchair users. Eur Sociol Rev. 2021;37(5):818–33.

[21] Shamshiri-Petersen D, Krogh C. Disability disqualifies: a vignette experiment of Danish employers’ intentions to hire applicants with physical disabilities. Scand J Disabil Res. 2020;22(1):198–209.

[22] Bendick M Jr. Employment discrimination against persons with disabilities: evidence from matched pair testing. Int J Divers Organ Communities Nations. 2018;17(1):11.

[23] McDonnall MC, Lund EM. Employers’ intent to hire people who are blind or visually impaired: a test of the theory of planned behavior. Rehabil Couns Bull. 2020;63(4):206–15.

[24] Papakonstantinou D, Papadopoulos K. Employers’ attitudes toward hiring individuals with visual impairments. Disabil Rehabil. 2020;42(6):798–805. 30636469 10.1080/09638288.2018.1510044

[25] Janssens KM, et al. Line managers’ hiring intentions regarding people with mental health problems: a cross-sectional study on workplace stigma. Occup Environ Med. 2021;78(8):593–9. 33542095 10.1136/oemed-2020-106955 PMC8292579

[26] Khayatzadeh-Mahani A, Wittevrongel K, Nicholas DB, Zwicker JD. Prioritizing barriers and solutions to improve employment for persons with developmental disabilities. Disabil Rehabil. 2020;42(19):2696–706. 30856355 10.1080/09638288.2019.1570356

[27] nTIDE Deeper Dive: Employment Trends for Disability Type-8/18/2023.

[28] Gewurtz RE, Langan S, Shand D. Hiring people with disabilities: a scoping review. Work. 2016;54(1):135–48. 26967030 10.3233/WOR-162265

[29] Shaw L, Daraz L, Bezzina MB, Patel A, Gorfine G. Examining macro and meso level barriers to hiring persons with disabilities: a scoping review. Environ Contexts Disabil. 2014;2:185–210.

[30] Nagtegaal R, de Boer N, van Berkel R, Derks B, Tummers L. Why do employers (fail to) hire people with disabilities? A systematic review of capabilities, opportunities and motivations. J Occup Rehabil. 2023;2:1–12. 10.1007/s10926-022-10076-1 PMC10172218 36689057

[31] Colella A, Varma A. Disability-job fit stereotypes and the evaluation of persons with disabilities at work. J Occup Rehabil. 1999;9:79–95.

[32] Dundon T, Wilkinson A. HRM in small and mediumsized enterprises (SMEs). In: Human resource management. London: Routledge; 2018. p. 194–211.

[33] Meinert MC. Opening doors. HR Magazine, vol. June 1, 2012. [Online]. Available: https://www.shrm.org/hr-today/news/hr-magazine/pages/0612meinert.aspx

[34] Schur L, Nishii L, Adya M, Kruse D, Bruyère SM, Blanck P. Accommodating employees with and without disabilities. Hum Resour Manage. 2014;53(4):593–621.

[35] Hyseni F, Kruse D, Schur L, Blanck P. Disability, workplace inclusion and organizational citizenship behavior: an exploratory study of the legal profession. J Particip Empl Ownersh. 2023;6(1):31–50. 10.1108/jpeo-10-2022-0017 PMC10961973 38528853

[36] Thomas A, Vaughn ED, Doyle A, Bubb R. Implicit association tests of attitudes toward persons with disabilities. J Exp Educ. 2014;82(2):184–204.

[37] Bureau of Labor Statistics. Economic news release table 4. Employed persons by disability status, industry, class of worker, and sex, 2023 annual averages. Feb. 22, 2024. Accessed: Mar. 28, 2024. [Online]. Available: https://www.bls.gov/news.release/disabl.t04.htm

[38] Conejo MA. At the intersection of feminist and disability rights movements. From equality in difference to human diversity claims. In: Disability and intersecting statuses. Bingley: Emerald Group Publishing Limited; 2013. p. 23–45.

[39] Frederick A, Shifrer D. Race and disability: from analogy to intersectionality. Sociol Race Ethn. 2019;5(2):200–14.

[40] Stienstra D. Troubling activisms: Canada and transnational disability activism. In: Global perspectives on disability activism and advocacy. London: Routledge; 2019. p. 298–314.

[41] Conner M, Norman P. Understanding the intention-behavior gap: the role of intention strength. Front Psychol. 2022;13: 923464. 35992469 10.3389/fpsyg.2022.923464 PMC9386038

[42] Araten-Bergman T. Managers’ hiring intentions and the actual hiring of qualified workers with disabilities. Int J Hum Resour Manag. 2016;27(14):1510–30.

