# Supplementary material for: Perceptions and Bias of Small Business Leaders in Employing People with Different Types of Disabilities
Source: J Occup Rehabil. Author manuscript; Available in PMC 2024 Jun 27. (PMC11180154; doi:10.1007/s10926-024-10201-2)
Supplement: Perceptions and Bias of Small Business Leaders in Employing People with Different Types of Disabilities Supplementary [file NIHMS1995133-supplement-Perceptions_and_Bias_of_Small_Business_Leaders_in_Employing_People_with_Different_Types_of_Disabilities_Supplementary.pdf]

*Table 1: Model 4a- Odds Ratio Logit, Companies with FEWER THAN 15 EMPLOYEES. Dependent Variable- Likelihood to Hire each type of disability, Independent variables include all standard controls and all Barriers and Facilitators**

	IDD	Blindness	Deafness	Physical	Mental Illness
Owner (versus manager)	2.509	0.001	0.141**	0.111*	0.126*
Pct Disability Employment	1.680	519.800	1.485	0.320	3.313
Years in Business	0.935*	0.859	0.956	0.968	0.938*
No Disability Relationships	0.426	0.641	0.421	0.667	0.610
Industry	1.000	1.000	1.000	1.000	1.000
Retail	9.105	113.600	1.296	0.366	0.275
Finance	4.527	470.500	2.361	1.950	1.220
Business	5.598	542.500	2.097	0.858	0.408
Other	12.850	0.001	5.153	0.0272**	1.000
Did not apply	5.210	22.490	0.590	6.467*	1.560
Not Qualified	1.535	1.246	0.853	0.725	9.068
Not familiar with how to accommodate	0.374	0.000	**13.44****	0.271	**0.0192***
Not as productive	0.212	0.000	**0.159***	**0.0179*****	6.659
Cannot Discipline	3.155	0.011	**0.0963***	1.259	**0.0178****
Uncertain about cost of accommodation	0.275	634.800	0.416	0.654	**8.003***
Fairness for other employees	1.513	2.106	3.649	4.019	0.500
Cost Outweigh benefits	0.104	16.990	0.456	2.436	0.242
Client/Customer reaction	2.000	1.738	2.489	0.114	6.010
Personal Familiarity	3.092	2.719	1.044	0.778	5.762
Improves Corporate Image	0.369	0.270	0.722	0.734	1.203
Positive Work Environment	2.185	0.803	1.162	4.946	1.156
Bottom Line	0.398	0.529	2.023	**15.41***	1.205
Company Policy	4.074	137.100	1.360	0.186	0.465
Costs versus Benefits	3.147	0.461	2.635	0.318	1.855
Government Policies	2.571	25.160	0.667	4.583	**0.0973***
_cons					
N	366	367	367	367	358

*The Barriers and Facilitators are binary variables indicating whether 1 indicates the respondent was at least somewhat concerned about a barrier and believed that the motivators were at least somewhat important. The Dependent variable is a binary variable indicating whether the respondent was very likely to hire a particular type of disability

Table 2: Model 3b- Odds Ratio Logit, Companies with 15 OR MORE EMPLOYEES. Dependent Variable- Likelihood to Hire each type of disability, Independent variables include all standard controls and all Barriers and Facilitators*

ADA=1					
	IDD	Blindness	Deafness	Physical	Mental Illness
Owner (versus manager)	0.932	0.501	0.219**	0.473	0.605
Pct Disability Employment	24.47	406.8	177.2	9724.1	32.83
Years in Business	0.985	0.965	0.997	0.992	0.941*
No Disability Relationships	0.637	0.902	0.159***	1.117	0.44
Industry					
Retail	15.11*	2.37	3.572	4.958	4.811
Finance	1.266	1.19	2.06	6.931	6.091
Business	4.635	5.267*	2.489	3.73	7.399
Other	5.826	1	2.372	2.951	5.974
Did not apply	1.925	1.995	3.124*	1.249	0.443
Not Qualified	2.734	0.579	1.674	2.262	2.959
Not familiar with how to accommodate	0.627	3.315	0.967	1.755	0.294
Not as productive	**0.214***	0.353	0.737	0.537	0.558
Cannot Discipline	0.397	**0.161***	**0.146****	0.298	0.414
Uncertain about cost of accommodation	0.304	0.148*	0.621	0.737	0.377
Fairness for other employees	0.446	1.812	1.477	0.656	2.791
Cost Outweigh benefits	6.904	2.972	0.845	**0.136***	1.931
Client/Customer reaction	1.077	0.214	0.459	0.641	0.493
Personal Familiarity	2.691	**30.42*****	2.773	3.322	1.975
Improves Corporate Image	2.347	0.574	0.273*	1.021	0.731
Positive Work Environment	0.285	0.0943	0.282	0.194	1.095
Bottom Line	0.839	0.5	13.17***	5.795*	0.725
Company Policy	0.759	1.734	0.372	0.83	0.618
Costs versus Benefits	**3.808***	0.169*	0.465	0.548	2.19
Government Policies	0.167**	3.771	2.918	0.635	0.792
_cons					
N	337	326	337	338	337
