# Supplementary material for: Perceptions and Bias of Small Business Leaders in Employing People with Different Types of Disabilities
Source: J Occup Rehabil. 2024 May 13;34(2):359–72. doi: 10.1007/s10926-024-10201-2 (PMC11180154; doi:10.1007/s10926-024-10201-2)
Supplement: Supplementary file 1 [file 10926_2024_10201_MOESM1_ESM.docx]

Table A1: Model 4a- Odds Ratio Logit, Companies with FEWER THAN 15 EMPLOYEES. Dependent Variable- Likelihood to Hire each type of disability, Independent variables include all standard controls and all Barriers and Facilitators*

|  | IDD | Blindness | Deafness | Physical | Mental Illness |
| --- | --- | --- | --- | --- | --- |
| Owner (versus manager) | 2.509 | 0.001 | 0.141** | 0.111* | 0.126* |
| Pct Disability Employment | 1.680 | 519.800 | 1.485 | 0.320 | 3.313 |
| Years in Business | 0.935* | 0.859 | 0.956 | 0.968 | 0.938* |
| No Disability Relationships | 0.426 | 0.641 | 0.421 | 0.667 | 0.610 |
| Industry | 1.000 | 1.000 | 1.000 | 1.000 | 1.000 |
| Retail | 9.105 | 113.600 | 1.296 | 0.366 | 0.275 |
| Finance | 4.527 | 470.500 | 2.361 | 1.950 | 1.220 |
| Business | 5.598 | 542.500 | 2.097 | 0.858 | 0.408 |
| Other | 12.850 | 0.001 | 5.153 | 0.0272** | 1.000 |
| Did not apply | 5.210 | 22.490 | 0.590 | 6.467* | 1.560 |
| Not Qualified | 1.535 | 1.246 | 0.853 | 0.725 | 9.068 |
| Not familiar with how to accommodate | 0.374 | 0.000 | **13.44**** | 0.271 | **0.0192*** |
| Not as productive | 0.212 | 0.000 | **0.159*** | **0.0179***** | 6.659 |
| Cannot Discipline | 3.155 | 0.011 | **0.0963*** | 1.259 | **0.0178**** |
| Uncertain about cost of accommodation | 0.275 | 634.800 | 0.416 | 0.654 | **8.003*** |
| Fairness for other employees | 1.513 | 2.106 | 3.649 | 4.019 | 0.500 |
| Cost Outweigh benefits | 0.104 | 16.990 | 0.456 | 2.436 | 0.242 |
| Client/Customer reaction | 2.000 | 1.738 | 2.489 | 0.114 | 6.010 |
| Personal Familiarity | 3.092 | 2.719 | 1.044 | 0.778 | 5.762 |
| Improves Corporate Image | 0.369 | 0.270 | 0.722 | 0.734 | 1.203 |
| Positive Work Environment | 2.185 | 0.803 | 1.162 | 4.946 | 1.156 |
| Bottom Line | 0.398 | 0.529 | 2.023 | **15.41*** | 1.205 |
| Company Policy | 4.074 | 137.100 | 1.360 | 0.186 | 0.465 |
| Costs versus Benefits | 3.147 | 0.461 | 2.635 | 0.318 | 1.855 |
| Government Policies | 2.571 | 25.160 | 0.667 | 4.583 | **0.0973*** |
| _cons |  |  |  |  |  |
| N | 366 | 367 | 367 | 367 | 358 |

*The Barriers and Facilitators are binary variables indicating whether 1 indicates the respondent was at least somewhat concerned about a barrier and believed that the motivators were at least somewhat important. The Dependent variable is a binary variable indicating whether the respondent was very likely to hire a particular type of disability

Table A2: Model 3b- Odds Ratio Logit, Companies with 15 OR MORE EMPLOYEES. Dependent Variable- Likelihood to Hire each type of disability, Independent variables include all standard controls and all Barriers and Facilitators*

| ADA=1 |  |  |  |  |  |
| --- | --- | --- | --- | --- | --- |
|  | IDD | Blindness | Deafness | Physical | Mental Illness |
| Owner (versus manager) | 0.932 | 0.501 | 0.219** | 0.473 | 0.605 |
| Pct Disability Employment | 24.47 | 406.8 | 177.2 | 9724.1 | 32.83 |
| Years in Business | 0.985 | 0.965 | 0.997 | 0.992 | 0.941* |
| No Disability Relationships | 0.637 | 0.902 | 0.159*** | 1.117 | 0.44 |
| Industry |  |  |  |  |  |
| Retail | 15.11* | 2.37 | 3.572 | 4.958 | 4.811 |
| Finance | 1.266 | 1.19 | 2.06 | 6.931 | 6.091 |
| Business | 4.635 | 5.267* | 2.489 | 3.73 | 7.399 |
| Other | 5.826 | 1 | 2.372 | 2.951 | 5.974 |
| Did not apply | 1.925 | 1.995 | 3.124* | 1.249 | 0.443 |
| Not Qualified | 2.734 | 0.579 | 1.674 | 2.262 | 2.959 |
| Not familiar with how to accommodate | 0.627 | 3.315 | 0.967 | 1.755 | 0.294 |
| Not as productive | **0.214*** | 0.353 | 0.737 | 0.537 | 0.558 |
| Cannot Discipline | 0.397 | **0.161*** | **0.146**** | 0.298 | 0.414 |
| Uncertain about cost of accommodation | 0.304 | 0.148* | 0.621 | 0.737 | 0.377 |
| Fairness for other employees | 0.446 | 1.812 | 1.477 | 0.656 | 2.791 |
| Cost Outweigh benefits | 6.904 | 2.972 | 0.845 | **0.136*** | 1.931 |
| Client/Customer reaction | 1.077 | 0.214 | 0.459 | 0.641 | 0.493 |
| Personal Familiarity | 2.691 | **30.42***** | 2.773 | 3.322 | 1.975 |
| Improves Corporate Image | 2.347 | 0.574 | 0.273* | 1.021 | 0.731 |
| Positive Work Environment | 0.285 | 0.0943 | 0.282 | 0.194 | 1.095 |
| Bottom Line | 0.839 | 0.5 | 13.17*** | 5.795* | 0.725 |
| Company Policy | 0.759 | 1.734 | 0.372 | 0.83 | 0.618 |
| Costs versus Benefits | **3.808*** | 0.169* | 0.465 | 0.548 | 2.19 |
| Government Policies | 0.167** | 3.771 | 2.918 | 0.635 | 0.792 |
| _cons |  |  |  |  |  |
| N | 337 | 326 | 337 | 338 | 337 |
